# Supplementary material for: KSHV promotes oncogenic FOS to inhibit nuclease AEN and transactivate RGS2 for AKT phosphorylation
Source: bioRxiv. 2024 Jan 28:2024.01.27.577582. Preprint. [Version 1] doi: 10.1101/2024.01.27.577582 (PMC10896338; doi:10.1101/2024.01.27.577582)

bioRxiv preprint doi: https://doi.org/10.1101/2024.01.27.577582; this version posted January 28, 2024. The copyright holder for this preprint (which was not certified by peer review) is the author/funder. This article is a US Government work. It is not subject to copyright under 17 USC 105 and is also made available for use under a CC0 license.

SUPPLEMENTAL INFORMATION

Table S1. KSHV ORF57 host targets in BCBL-1 cells identified by ORF57 CLIP-seq (this study) and ORF57 HITS-CLIP (Sei et al., 2015, PLoS Pathog 11(2): e1004652; doi.org/10.1371/journal.ppat.1004652).

Table S2. Peaks representing ORF57-binding sites in transcripts of host protein-coding genes identified by Piranha software based on anti-ORF57 CLIP-seq as shown in Fig.

bioRxiv preprint doi: https://doi.org/10.1101/2024.01.27.577582; this version posted January 28, 2024. The copyright holder for this preprint (which was not certified by peer review) is the author/funder. This article is a US Government work. It is not subject to copyright under 17 USC 105 and is also made available for use under a CC0 license.

1C. The associated p-value represents zero-truncated negative binomial distribution. The peaks with a p-value less than 0.05 are considered significant.

Table S3. Differential expression of host coding and noncoding genes in BCBL-1 cells with KSHV latent versus lytic infection.

Table S4. Differential expression of coding and noncoding genes in HEK293T cells without versus with KSHV ORF57.

Table S5. AP-1 sensitive genes identified by GSEA from BCBL-1 cells with KSHV lytic infection.

Table S6. Oligonucleotides used in the study.

Supplemental Figures

Figure S1. ORF57 regulates the expression of host protein-coding RNAs. (A and B) Principal component (PC) analysis of RNA-seq of BCBL-1 cells with latent or lytic infection (A) and HEK293T cells without (ctrl) or with ORF57 expression (B); each point represents individual samples in each group. (C and D) Volcano plots of differentially expressed host genes in BCBL-1 cells with lytic versus latent infection (C) and HEK293T cells with ORF57 expression over the control cells without ORF57 (D). Genes significantly ($p<0.001$) upregulated in FC (fold change ≥ 2) are shown as red dots and downregulated FC ≤ -2 as blue dots. (E) Validation of interaction between ORF57 and SOCS3, EMP3, and FASN RNA by RIP. RT-PCR in the presence (+) or absence (-) of RT with a gene-specific primer set (Table 1) was performed on RNA

bioRxiv preprint doi: https://doi.org/10.1101/2024.01.27.577582; this version posted January 28, 2024. The copyright holder for this preprint (which was not certified by peer review) is the author/funder. This article is a US Government work. It is not subject to copyright under 17 USC 105 and is also made available for use under a CC0 license.

immunoprecipitated (RIP) from BCBL-1 cells during KSHV lytic infection by anti-ORF57 antibody. Corresponding non-specific IgG served as a negative control.

Figure S2. FOS expression is not regulated by ORF57 at the translation level. A) A representative gel of two in vitro transcription/translation assays using rabbit reticulocyte lysates in the presence of ^{35}S-methionine. *FOS* cDNA under the T7 promoter was used as a template. The recombinant FLAG-tagged ORF57 protein was added at increasing concentrations (5-50 nM) in the reaction and incubated at 30 °C for 1.5 h. The reaction was stopped by the addition of 2 × LDS/2-ME protein sample buffer and heated at 80 °C for 10 min. ^{35}S-met-labeled proteins were resolved in SDS-PAGE gel and captured by phosphorimager screen or X-ray film. Firefly luciferase RNA (Luc) was included as a negative control.

Figure S3. FOS expression is sensitive to host MTR4. (A) MTR4 knockdown (KD) by MTR4-targeting siRNA (siMTR4) promotes FOS protein expression in HEK293T cells. The effect of MTR4 KD on FOS expression in HEK293T cells was examined by Western blotting at 48 h after transfection. A non-targeting siRNA (siNT) served as a siRNA control and GAPDH as a sample loading control. (B) KSHV lytic infection and ORF57 expression do not affect MTR4 expression. MTR4 RNA expression was quantified by RT-qPCR in BCBL-1 with latent and lytic infection and in HEK293T cells transfected with an ORF57-expressing (ORF57) or an empty vector control (Ctrl) vector. Data from all three separate experiments, each with three replicates, were averaged with mean ± SD. NS – no significance, two-tailed Student *t*-test.

bioRxiv preprint doi: https://doi.org/10.1101/2024.01.27.577582; this version posted January 28, 2024. The copyright holder for this preprint (which was not certified by peer review) is the author/funder. This article is a US Government work. It is not subject to copyright under 17 USC 105 and is also made available for use under a CC0 license.

(C) No protein-protein interaction between ORF57 and MTR4. Co-immunoprecipitation of ORF57 and MTR4 proteins from total cell extracts from HEK293T cells transfected with an ORF57-expressing vector was performed by using rabbit anti-ORF57 or anti-MTR4 antibodies, with corresponding species-specific IgG isotype serving as an antibody control. ORF57 and MTR4 proteins were immunoblotted with the corresponding antibodies as indicated.

bioRxiv preprint doi: https://doi.org/10.1101/2024.01.27.577582; this version posted January 28, 2024. The copyright holder for this preprint (which was not certified by peer review) is the author/funder. This article is a US Government work. It is not subject to copyright under 17 USC 105 and is also made available for use under a CC0 license.

Figure S1

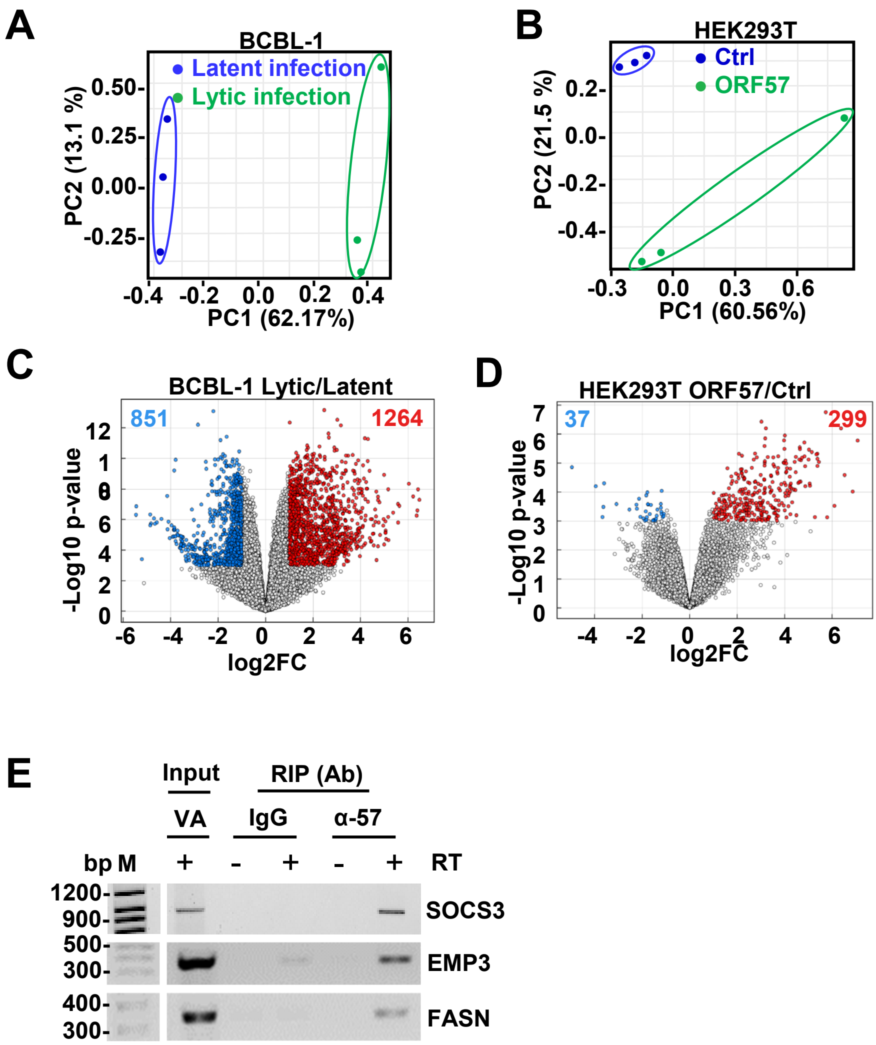

bioRxiv preprint doi: https://doi.org/10.1101/2024.01.27.577582; this version posted January 28, 2024. The copyright holder for this preprint (which was not certified by peer review) is the author/funder. This article is a US Government work. It is not subject to copyright under 17 USC 105 and is also made available for use under a CC0 license.

Figure S2

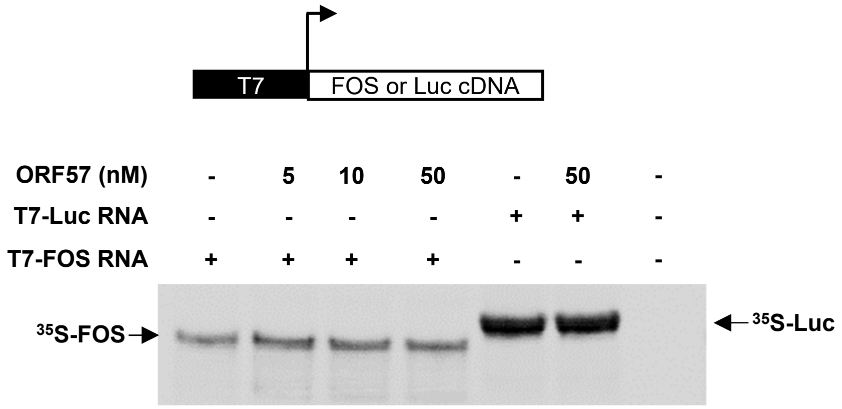

bioRxiv preprint doi: https://doi.org/10.1101/2024.01.27.577582; this version posted January 28, 2024. The copyright holder for this preprint (which was not certified by peer review) is the author/funder. This article is a US Government work. It is not subject to copyright under 17 USC 105 and is also made available for use under a CC0 license.

Figure S3

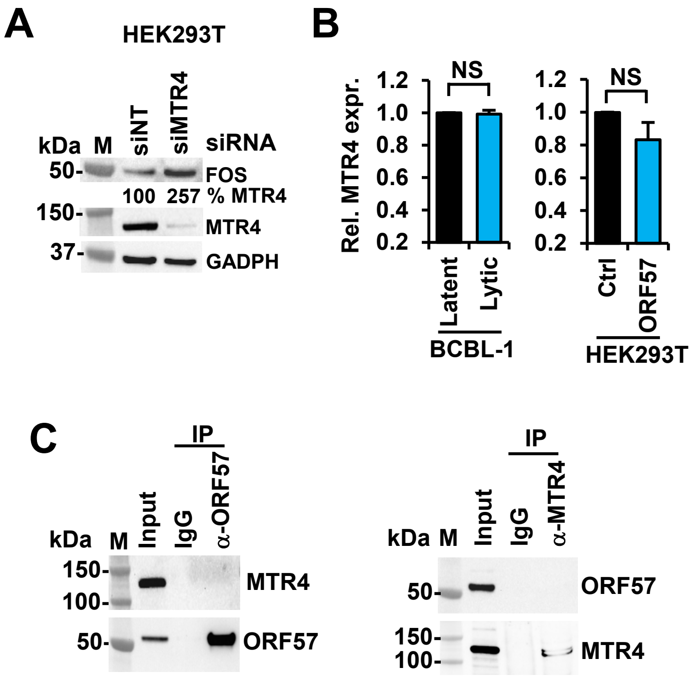

Supplement: Supplement 7 [file NIHPP2024.01.27.577582v1-supplement-7.pdf]
